# Hepatitis B reactivation among 1962 patients with hematological malignancy in Taiwan

**DOI:** 10.1186/s12876-017-0735-1

**Published:** 2018-01-08

**Authors:** Chien-Yuan Chen, Feng-Ming Tien, Aristine Cheng, Shang-Yi Huang, Wen-Chien Chou, Ming Yao, Jih-Luh Tang, Hwei-Fang Tien, Wang-Huei Sheng

**Affiliations:** 1 Division of Hematology, Department of Internal Medicine, National Taiwan University Hospital, Taipei, Taiwan; 2 Division of Infectious Disease, Department of Internal Medicine, National Taiwan University Hospital, 7 Chung-Shan South Road, Taipei, 10002 Taiwan; 3 Department of Laboratory Medicine, National Taiwan University Hospital, Taipei, Taiwan; 4 Tai-Cheng Stem Cell Therapy Center, National Taiwan University Hospital, Taipei, Taiwan

**Keywords:** Hepatitis B reactivation, Hematological malignancy, Antiviral prophylaxis, Chemotherapy

## Abstract

**Background:**

The risk of Hepatitis B virus (HBV) reactivation in patients with different hematological malignancy except lymphoma were rarely known before.

**Methods:**

A total of 1962 patients with hematological malignancy were enrolled and followed-up at the National Taiwan University Hospital between 2008 and 2013. The clinical characteristics, HBV serology, and laboratory data were retrospectively reviewed and analyzed.

**Results:**

A total of 1962 patients comprising 1048 men and 914 women were studied. The median age of the patients was 55 years (range, 15–97 years). Chronic HBV carriage was documented at diagnosis of hematological malignancy in 286 (14.6%) patients. A total of 171 (59.8%) of the 286 HBV carriers received primary prophylaxis with anti-HBV agents. Of the HBV carriers, 97 (33.9%) developed hepatitis B reactivation during or after chemotherapy, including 59 patients who had discontinued antiviral therapy. The incidence of hepatitis B reactivation among patients with hematological malignancy and HBV carriage was 10.4 per 100 person–years. A multivariate analysis revealed hepatocellular carcinoma (*p* < 0.001) and antiviral prophylaxis use (*p* < 0.001) were independent risk factors of HBV reactivation in HBV carriers. Of the 1676 patients with initial negative hepatitis B surface antigen (HBsAg) counts, 41 (2.4%) experienced hepatitis B reactivation, reverse seroconversion of HBsAg, and lost their protective hepatitis B surface antibody (anti-HBs). A multivariate analysis revealed that diabetes mellitus (*p* = 0.005, odds ratio (OR): 0.218, 95% confidence interval (CI): 0.076–0.629), allogeneic transplantation (*p* = 0.013, OR: 0.182, 95% CI: 0.047–0.701), liver cirrhosis (*p* < 0.001, OR: 0.002, 95% CI: 0–0.047), low anti-HBs titers (*p* = 0.016, OR: 0.020, 95% CI: 0.001–0.480), and positive hepatitis B core antibody (*p* = 0.013, OR: 0.070, 95% CI: 0.009–0.571) were independent risk factors of positive seroconversion of HBsAg in patients with hematological malignancy.

**Conclusions:**

The incidence of HBV reactivation among the patients with varying subtypes of hematological malignancy is similar. Prophylaxis with anti-HBV agents critically reduced the risk of hepatitis B reactivation.

## Background

Hepatitis B virus (HBV) is a DNA virus transmitted parenterally, sexually, and perinatally. HBV affects 350 to 400 million persons worldwide and constitutes a major global health burden [1, 2]. HBV infection can cause acute and chronic liver diseases including cirrhosis and hepatocellular carcinoma (HCC). Following immunosuppression, HBV replication along with signs of hepatocellular injury in a silent hepatitis B surface antigen (HBsAg) carrier may occur [3]. The clinical presentation of HBV reactivation ranges from asymptomatic to severe fulminant hepatitis, liver failure, and death. HBV reactivation in cancer patients undergoing cytotoxic chemotherapy has been observed in patients with lymphoma [4, 5], patients treated with corticosteroids [6, 7] and rituximab [8, 9], as well as in patients undergoing stem cell and bone marrow transplantation [10, 11]. HBV has long been endemic in Taiwan, and, previously, the seropositive rates of hepatitis B core antibody (anti-HBc) approached 80%–90% and the carrier rate of HBsAg in the population reached a level as high as 15%–20% prior to the nationwide hepatitis B vaccination program [12–14].

Hematological malignancy is a group of clonal blood disorders that affect lymphoid, myeloid, and stem cells. The clinical manifestations of hematological malignancy usually present with anemia, thrombocytopenia, leukopenia, and varying degrees of immunocompromised status. Conventional cytotoxic chemotherapy is the major treatment for hematological malignancy. Immunotherapy, graft versus host disease, immunosuppressant, and stem cell transplantation also detract from immune functions and result in HBV reactivation [4–11]. Reactivation of viral hepatitis, including hepatitis B, is a potential cause for abnormal liver function tests in leukemic patients receiving chemotherapy [15, 16]. Various chemotherapy agents and steroids are frequently used as part of a combination regimen for hematological malignancy. HBV reactivation has been highlighted as being crucial for the treatment of lymphoma [4–6]. Several comprehensive guidelines have been proposed to provide evidence-based recommendations for clinicians caring for this patient population [17–22]. However, the epidemiology and clinical risk factors among different subtypes of hematological malignancy remain unclear. We previously investigated the epidemiology and clinical manifestations of hepatitis B reactivation among 490 acute myeloid leukemia (AML) patients [23]. The incidence of hepatitis B reactivation among AML patients with HBV carriage was 9.5 per 100 person–years [23]. Hepatitis B reactivation is not uncommon in HBsAg positive AML patients. In the present study, we investigated the epidemiology of HBV reactivation for varying subtypes of hematological malignancy and analyzed the risk factors among patients with different subtypes of hematological malignancy.

## Methods

### Hospital setting and patients

National Taiwan University Hospital (NTUH) is a 2900-bed teaching hospital in Taipei metropolitan area. The clinical characteristics, hepatitis B serology, hepatitis B virus DNA data, and outcome results of all adult patients with hematological malignancy during the period of January 2008 to December 2013 that attended NTUH were retrospectively reviewed and analyzed. Liver function tests, hepatitis B serology titer, and HBV DNA levels were performed as clinically indicated. This study was approved by the National Taiwan University Hospital Institutional Review Board.

### Definitions

The biochemistry alanine aminotransferase (ALT) was measured using the Beckman Coulter AU5800 platform (Beckman Coulter Inc., Brea, CA, USA); HBsAg, hepatitis B surface antibody (anti-HBs), anti-HBc, hepatitis B envelope antigen (HBeAg), and hepatitis C antibody (anti-HCV) were measured using the Abbott ARCHITECT i2000SR (Abbott Laboratories, Abbott Park, North Chicago, IL, USA); HBV DNA was analyzed using the COBAS® AmpliPrep /COBAS® TaqMan® HBV Test, v2.0 (Roche, Basel, Switzerland) in accordance with manufacturer instructions. The serological results were defined by the following cut-off values: HBsAg positive was ≧0.05 IU/mL; anti-HBs positive was a seroprotection threshold of 10mIU/ mL; and anti-HBc positive was ≧1.0 IU/mL. HBeAg positive was defined as ≧1.0 IU/mL. Chronic hepatitis B carrier status was defined by the detection of positive HBsAg counts for more than 6 months. Hepatitis B reactivation was defined as a greater than 10-fold increase, compared with previous nadir levels of HBV DNA, or by the reappearance of HBeAg in the serum for patients whose baseline HBeAg count was negative [5, 6]. HBV-related hepatitis was defined as a greater than 3-fold increase of the serum alanine aminotransferase (ALT) level (the upper normal limit is 41 IU/L at NTUH) accompanying or following HBV reactivation. In July 1984, the Taiwan government launched a nationwide universal HBV vaccination program [13]. The following catch-up program of the nationwide HBV vaccination had a coverage rate of 86.9% to 98.0% [14].

The diagnosis of cirrhosis was based on clinical symptoms and signs and imaging studies (ultrasound and computed tomography). The diagnostic criteria of HCC in this study were based on histologic and/or clinical findings and on the presence of the following: chronic viral hepatitis infection and liver cirrhosis, hepatic tumor with imaging (ultrasound and computed tomography) characteristics compatible with a diagnosis of HCC and without evidence of gastrointestinal or other primary tumor, and a persistent elevation of the serum level of alpha-fetoprotein to 400 ng/mL or higher.

### Statistical analysis

The survival rates were estimated using the Kaplan-Meier analysis and compared using the log-rank test. Categorical variables were compared using the chi-square test. Univariate and multivariate analyses with a Cox regression analysis (log-rank test) were performed. Factors with *p*-values of ≤0.2 in univariate analysis were used in the multivariate model. All statistical analyses were performed using the statistical package SPSS for Windows v.18 (SPSS Inc., Chicago, IL). A *p*-value of ≤0.05 was considered significant and all statistical tests were two-tailed.

## Results

### Epidemiology

**Fig. 1:**
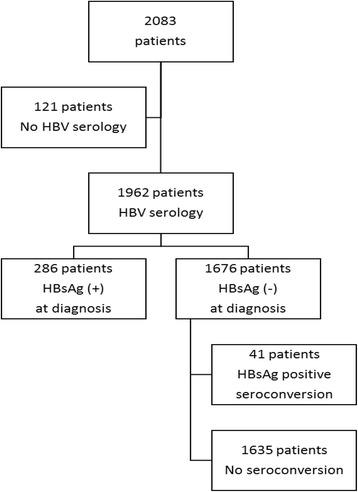
Study flow and hepatitis B serological data of patients at diagnosis of hematological malignancy

There were 2083 patients with hematological malignancy admitted to the hematological ward of the National Taiwan University Hospital between 2008 and 2013. The flow chart is illustrated in Fig. 1. A total of 1962 patients with hepatitis B serological profile at diagnosis of hematological malignancy were investigated, including 1046 men and 914 women. The median age was 55 years (range 15–97 years). The detailed clinical characteristics and laboratory data are listed in Table 1. The most common diagnosis of hematological malignancy was lymphoma, with 769 people (39.2%), followed by acute myeloid leukemia (AML), with 606 people (30.9%), myeloma, with 197 people (10.0%), acute lymphoblastic leukemia (ALL), with 160 people (8.2%), myelodysplastic syndrome (MDS) or aplastic anemia (AA), with 142 people (7.2%), chronic myeloid leukemia (CML) or myeloproliferative neoplasm (MPN), with 60 people (3.1%), and chronic lymphocytic leukemia (CLL), with 27 people (1.4%). Of 1962 patients, 286 (14.6%) were diagnosed with positive HBsAg during their diagnosis of hematological malignancy. There were 174 of 286 HBsAg-positive patients had HBeAg data at diagnosis of malignancy. Positive Hbe and anti-Hbe rate were 21.3% (37 / 174) and 80.4% (107 / 133) in 286 HBV carriers. A total of 97 (33.9%) of 286 HBsAg-positive patients had HBV reactivation during follow-up. 71 (73.2%) of 97 patients with HBV reactivation developed hepatitis and the peak median alanine aminotransferase level is 613 IU/L (range 105–3506 IU/L). Ten patients died due to fulminant hepatitis and hepatic failure. For the median follow-up of 1050 days, the incidence of HBV reactivation in 286 HBV carriers was 10.4 per 100 person–years. The higher incidence value was 13.4 per 100 person–years for AML, and the lower was 7.4 per 100 person-years for CML/MPN, and there was no difference among people with varying subtypes of hematological malignancy (*p* = 0.335).

**Table 1:** Clinical and laboratory data of patients with hematological malignancy

		Total	AML	ALL	Lymphoma	CML/MPN	CLL	MM	MDS/AA	*p*
Number		1962	606	160	769	60	27	197	143	
Gender	Man	1048	326	83	399	36	16	108	81	0.799
	Woman	914	280	77	370	24	11	89	62	
Age, median(range), year		55(15–97)	54(17–92)	38(16–86)	57(15–97)	48(18–86)	61(37–89)	59(30–87)	55(17–94)	<0.001
HBs Ag(+) at diagnosis		286	75	13	142	6	5	31	14	0.002
HBV reactivation		97	29	3	46	1	2	10	6	0.335
HBs Ag(−) positive seroconversion		41	5	7	24	0	1	3	1	0.013
Hepatitis C		73	17	0	42	1	1	5	7	0.013
Liver cirrhosis		21	6	1	10	1	0	1	2	0.924
Hepatocellular carcinoma		9	3	0	4	2	0	0	0	0.038
Underlying diabetes		273	75	22	109	8	4	31	24	0.833
Allogeneic transplantation		439	233	81	48	18	2	3	54	<0.001

*AML* acute myeloid leukemia, *ALL* acute lymphoblastic leukemia, *CML/MPN* chronic myeloid leukemia/myeloproliferative neoplasm, *CLL* chronic lymphocytic leukemia, *MM* myeloma, *MDS/AA* myelodysplastic syndrome/aplastic anemia

A total of 41 (2.4%) of 1676 patients whose HBsAg was negative initially displayed positive seroconversion during subsequent follow–ups and had their HBsAg become positive. Anti-HBc data existed for a total of 849 patients, and 567 (66.8%) patients showed positive anti-HBc in this cohort.

### Prophylaxis of hepatitis B reactivation in patients with hematological malignancy

There were 286 patients with positive HBsAg at diagnosis of hematological malignancy. Of the 286 patients, the primary prophylaxis for 171 patients (59.8%) was antiviral agents, including 85 (49.7%) patients with entecavir, 76 (44.4%) with lamivudine, 2 (1.2%) with telbivudine, 1 (0.6%) with tenofovir, and 7 (4.1%) with adefovir. A total of 97 patients had HBV reactivation and 71 (73.2%) of the 97 patients had hepatitis. There were 59 patients who developed HBV reactivation after a discontinuance period of antiviral agent primary prophylaxis, and the median length for HBV reactivation was 210 days (range 15–2349 days) after discontinuing the antiviral drug. Even under antiviral drug use, 26 patients had HBV reactivation, including 6 who had tyrosine–methionine–aspartate– aspartate mutation. There were 12 patients who had HBV reactivation without antiviral prophylaxis. A total of 10 (10.3%) out of 97 HBV reactivation patients died of fulminant hepatitis and hepatic failure, including 5 patients who had discontinued use of the antiviral drug (median period after discontinuance was 96 days, range 44–226 days), 3 patients who did not use antiviral prophylaxis, and 2 other patients with HBV reactivation even with use of antiviral prophylaxis.

### Risk of HBV reactivation in HBV carrier

We analyzed the risk factors of HBV reactivation in 286 HBsAg positive patients. The detailed results are listed in Table 2. The variables comprised hematological malignancy subtypes, age, gender, concurrent hepatitis C infection, liver cirrhosis, hepatocellular carcinoma, diabetes mellitus, allogeneic transplantation recipient, and antiviral prophylaxis use. A univariate analysis revealed that hepatocellular carcinoma (*p* = 0.032) and antiviral prophylaxis (*p* < 0.001) were risk factors of HBV reactivation in an HBV carrier, and age > 65 years was associated with a reduced rate of HBV reactivation (*p* = 0.052). A multivariate analysis revealed that both hepatocellular carcinoma (*p* < 0.001, odds ratio (OR): 0.101, 95% confidence interval (CI): 0.029–0.355), and antiviral prophylaxis (*p* < 0.001, OR: 4.631, 95% CI: 2.902–7.390) were independent risk factors of HBV reactivation in HBsAg-positive patients.

**Table 2:** Risk factors of HBV reactivation in HBsAg-positive patients with hematological malignancy

Factors	Reactivation *N* = 97	No Reactivation *N* = 189	Univariate *P* value	Multivariate *P* Value	Odds ratio(95% CI)
Hematological malignancy			0.332	NA	
AML	29	46			
ALL	3	10			
Lymphoma	46	96			
CML/MPN	1	5			
CLL	2	3			
Myeloma	10	21			
MDS/AA	6	8			
Age > 65 year			0.052	0.198	
Yes	11	57			
No	86	132			
Gender			0.781	NA	
Man	55	106			
Woman	42	83			
Hepatitis C			0.180	0.453	
Yes	4	4			
No	93	185			
Liver cirrhosis			0.906	NA	
Yes	4	7			
No	93	182			
Hepatocellular carcinoma			0.032	<0.001	0.101(0.029–0.355)
Yes	3	1			
No	94	188			
Diabetes mellitus			0.388	NA	
Yes	10	20			
No	87	169			
Allogeneic transplantation			0.113	0.269	
Yes	28	31			
No	69	158			
Antiviral prophylaxis			<0.001	<0.001	4.631(2.902–7.390)
Yes	26	145			
No	71	44			

*AML* acute myeloid leukemia, *ALL* acute lymphoblastic leukemia, *CML/MPN* chronic myeloid leukemia/myeloproliferative neoplasm, *CLL* chronic lymphocytic leukemia, *MM* myeloma, *MDS/AA* myelodysplastic syndrome/aplastic anemia

### Risk factor of hepatitis B reverse seroconversion

We observed that 41 (2.4%) out of the 1676 patients who were HBsAg negative at diagnosis of hematological malignancy exhibited reverse seroconversion to HBsAg positive in the follow-up, including 7 (4.8%) out of 147 patients with ALL, 1 (4.5%) out of 22 with CLL, 24 (3.8%) out of 627 with lymphoma, 5 (0.9%) out of 531 with AML, 3 (1.8%) out of 166 with myeloma, and 1 (0.8%) out of 129 with MDS and AA, respectively. There were 54 patients with CML/MPN; however, out of them, no one acquired reverse seroconversion. The median follow–up period was 835 days (range 1–2976 days). A total of 36 (87.8%) out of 41 patients had hepatitis after reverse seroconversion, and 1 patient died of fulminant hepatitis and hepatic failure. The detailed results are provided in Table 3.

**Table 3:** Risk factors for positive seroconversion of HBsAg in HBsAg-negative patients detected during diagnosis of hematological malignancy

	Positive Sero- conversion (*n* = 41)	No conversion (*n* = 1635)	Univariate *P* value	Multivariate *P* value	Odds ratio (95% CI)
Age			0.174	NA	
≧65 years	12	466			
< 65 years	29	1169			
Gender			0.737	NA	
Men	22	866			
Women	19	769			
Hematological malignancy			0.038	0.084	NA
AML	5	526			
ALL	7	140			
Lymphoma	24	603			
CML/MPN	0	54			
CLL	1	21			
Myeloma	3	163			
MDS / AA	1	128			
Diabetes mellitus			0.040	0.005	0.218(0.076–0.629)
Yes	10	233			
No	31	1402			
Allogeneic transplantation			0.116	0.013	0.182(0.047–0.701)
Yes	15	365			
No	26	1270			
Liver cirrhosis			<0.001	<0.001	0.002(0.000–0.047)
Yes	2	8			
No	39	1627			
Hepatocellular carcinoma			0.748	NA	
Yes	0	5			
No	41	1630			
Hepatitis C			0.216	NA	
Yes	0	65			
No	41	1570			
Negative Anti-HBs Antibody^a^			0.237	NA	
Yes	10	304			
No	24	1157			
Low Anti-HBs Antibody ^a^			0.002	0.016	0.020(0.001–0.480)
Yes	30	915			
No	4	546			
Positive Anti-HBc Antibody^b^			0.005	0.013	0.070(0.009–0.571)
Yes	18	567			
No	1	263			

*NA* not available, ^a^ Anti-HBs Antibody checked, (*n* = 1495), Low Anti-HBs Antibody <100mIU/mL, ^b^ Anti-HBc Antibody checked (*n* = 849), *AML* acute myeloid leukemia, *ALL* acute lymphoblastic leukemia, *CML/MPN* chronic myeloid leukemia/myeloproliferative neoplasm, *CLL* chronic lymphocytic leukemia, *MM* myeloma, *MDS/AA* myelodysplastic syndrome/aplastic anemia

We analyzed the risk of reverse seroconversion in 1676 patients with hematological malignancy. The variables comprised hematological malignancy subtypes, age, gender, concurrent hepatitis C infection, liver cirrhosis, hepatocellular carcinoma, diabetes mellitus, allogeneic transplantation recipient, anti-HBs, and anti-HBc. A univariate analysis revealed that hematological subtypes (*p* = 0.038), diabetes mellitus (*p* = 0.04), liver cirrhosis (*p* < 0.001), low anti-HBs titer values (*p* = 0.002), and positive anti-HBc (*p* = 0.005) were risk factors of HBsAg positive seroconversion. A multivariate analysis revealed that diabetes mellitus (*p* = 0.005, OR: 0.218, 95% CI: 0.076–0.629), allogeneic transplantation (*p* = 0.013, OR: 0.182, 95% CI: 0.047–0.701), liver cirrhosis (*p* < 0.001, OR: 0.002, 95% CI: 0.000–0.047), low anti-HBs titer values (*p* = 0.016, OR: 0.020, 95% CI: 0.001–0.480), and positive anti-HBc (*p* = 0.013, OR: 0.070, 95% CI: 0.009–0.571) were independent risk factors of HBsAg positive seroconversion. Having different subtypes of hematological malignancy had no impact on HBsAg positive seroconversion in patients.

## Discussion

HBV is endemic in Taiwan. Mass vaccination started in 1984 [13, 14]. However, the prevalence of HBV is still high in the general population. This is the first large cohort study to compare the incidence of HBV reactivation in different subtypes of hematological malignancy. In this retrospective cohort, 286 (14.6%) of 1962 patients were HBV carriers. HBV reactivation is critical for the clinical care of patients with hematological malignancy receiving chemotherapy. The incidence of HBV reactivation was 10.4 per 100 person–years in this study. This epidemiological result is similar to those of previous studies of lymphoma (10.4 per 100 person–years) [5] and AML (9.5 per 100 person–years) [23].

HBV reactivation in cancer patients receiving cytotoxic chemotherapy has been noted for 3 decades [3, 24, 25], especially in patients with lymphoma [4, 5], patients treated with corticosteroids [6, 7] and rituximab [8, 9], as well as in patients undergoing stem cell and bone marrow transplantation [10, 11]. There have been some reports concerning HBV reactivation in patients with AML [23, 26, 27] and myeloma [28, 29]. We observed HBV reactivation in patients with different subtypes of hematological malignancy. This study confirmed that HBV reactivation developed in patients with different subtypes of hematological malignancy. Antiviral drug prophylaxis is critical for HBV reactivation according to most guidelines and professional consensuses [17–22]. In the studied cohort, 59 (60.8%) out of 97 HBV carriers experienced HBV reactivation after discontinuing use of antiviral medication. Thus, the ideal length that HBV patients should use antiviral medication for after chemotherapy is still a controversial topic.

Resolved hepatitis B reactivation and acquired reverse seroconversion has been investigated in recent literature [30–33]. In one such study, 33 (6.8%) out of 482 lymphoma patients acquired HBV reverse seroconversion between 2000 and 2010 [33]. HBV reverse seroconversion patients typically receive more cycles (≥6), prolonged durations of rituximab therapy, and hematopoietic stem cell transplantation than non-HBV reverse seroconversion patients do. In the present studied cohort, we observed that 41 (2.4%) of out 1676 patients acquired HBV reverse seroconversion. There were 54 patients with CML/MPN; however, out of these patients no one acquired reverse seroconversion. We also observed that HBV reverse seroconversion could be observed in patients with other subtypes of hematological malignancy other than lymphoma. Diabetes mellitus, allogeneic transplantation, liver cirrhosis, low anti-HBs titers (less than 100 mI U/mL), and positive anti-HBc were independent risk factors of HBV reverse seroconversion.

Diabetes mellitus is an immunocompromised status and may trigger spontaneous HBV reactivation [34–36]. The anti-HBc positive rate was 69% in this study. All patients with HBV reverse seroconversion displayed negative anti-HBc serology. Because the rate of HBV reverse seroconversion was 2.4% in this cohort, treating all resolved HBV patients receiving chemotherapy in Taiwan does not seem likely. The high risk factors of a stratified risk comprise diabetes mellitus, allogeneic transplantation, liver cirrhosis, low anti-HBs titers (less than 100 mIU/mL), and positive anti-HBc. Thus, predicting the risk of HBV reverse seroconversion is possible.

## Conclusions

The treatment of hematological malignancy has improved during the past several decades. Conventional cytotoxic chemotherapy, rituximab-based immunotherapy, and stem cell transplantation can cause a patient to become immunocompromised and result in HBV reactivation. We observed that HBV carriers and patients with resolved HBV could experience reverse seroconversion and reactivation. The incidence of HBV reactivation did not differ significantly among various subtypes of hematological malignancy. Antiviral agent prophylaxis is the most critical method to prevent HBV reactivation. However, some patients develop HBV reactivation after discontinuing use of antiviral agents after chemotherapy. Thus, the duration of antiviral agent treatment after chemotherapy should be further investigated.

## Abbreviations

AA: Aplastic anemia
ALL: Acute lymphoblastic leukemia
ALT: Alanine aminotransferase
AML: Acute myeloid leukemia
Anti-HBc: Anti-hepatitis B core antibody
Anti-HBs: Anti-hepatitis B surface antigen antibody
Anti-HCV: Anti-hepatitis C virus antibody
CI: Confidence interval
CLL: Chronic lymphocytic leukemia
CML: Chronic myeloid leukemia
HBeAg: Hepatitis B envelope antigen
HBsAg: Hepatitis B surface antigen
HBV: Hepatitis B virus
HCC: Hepatocellular cell carcinoma
MDS: Myelodysplastic syndrome
MPN: Myeloproliferative neoplasm
OR: Odds ratio

## References

[1] Dienstag JL (2008). Hepatitis B virus infection. N Engl J Med.

[2] Trépo C, Chan HL, Lok A (2014). Hepatitis B virus infection. Lancet.

[3] Shouval D, Shibolet O (2013). Immunosuppression and HBV reactivation. Semin Liver Dis.

[4] Yeo W, Chan PK, Zhong S, Ho WM, Steinberg JL, Tam JS (2000). Frequency of hepatitis B virus reactivation in cancer patients undergoing cytotoxic chemotherapy: a prospective study of 626 patients with identification of risk factors. J Med Virol.

[5] Hsu C, Tsou HH, Lin SJ, Wang MC, Yao M, Hwang WL (2014). Chemotherapy-induced hepatitis B reactivation in lymphoma patients with resolved HBV infection: a prospective study. Hepatology.

[6] Cheng AL, Hsiung CA, Su IJ, Chen PJ, Chang MC, Tsao CJ (2003). Steroid-free chemotherapy decreases risk of hepatitis B virus (HBV) reactivation in HBV-carriers with lymphoma. Hepatology.

[7] Yeo W, Zee B, Zhong S, Chan PK, Wong WL, Ho WM (2004). Comprehensive analysis of risk factors associating with hepatitis B virus (HBV) reactivation in cancer patients undergoing cytotoxic chemotherapy. Br J Cancer.

[8] Kusumoto S, Tanaka Y, Ueda R, Mizokami M (2011). Reactivation of hepatitis B virus following rituximab-plus-steroid combination chemotherapy. J Gastroenterol.

[9] Tsutsumi Y, Yamamoto Y, Shimono J, Ohhigashi H, Teshima T (2013). Hepatitis B virus reactivation with rituximab-containing regimen. World J Hepatol.

[10] Martin BA, Rowe JM, Kouides PA, DiPersio JF (1995). Hepatitis B reactivation following allogeneic bone marrow transplantation: case report and review of the literature. Bone Marrow Transplant.

[11] Liang R, Lau GK, Kwong YL (1999). Chemotherapy and bone marrow transplantation for cancer patients who are also chronic hepatitis B carriers: a review of the problem. J Clin Oncol.

[12] Hsu HM, Lu CF, Lee SC, Lin SR, Chen DS (1999). Seroepidemiologic survey for hepatitis B virus infection in Taiwan: the effect of hepatitis B mass immunization. J Infect Dis.

[13] Chang MH, Chen CJ, Lai MS, Hsu HM, Wu TC, Kong MS (1997). Universal hepatitis B vaccination in Taiwan and the incidence of hepatocellular carcinoma in children. Taiwan childhood Hepatoma study group. N Engl J Med.

[14] Ni YH, Huang LM, Chang MH, Yen CJ, Lu CY, You SL (2007). Two decades of universal hepatitis B vaccination in Taiwan: impact and implication for future strategies. Gastroenterology.

[15] Ishiga K, Kawatani T, Suou T, Tajima F, Omura H, Idobe Y (2001). Fulminant hepatitis type B after chemotherapy in a serologically negative hepatitis B virus carrier with acute myelogenous leukemia. Int J Hematol.

[16] Wong GC, Tan P, Goh YT, Ng HS, Chong R, Lee LH (1996). Exacerbation of hepatitis in hepatitis B carriers following chemotherapy for haematological malignancies. Ann Acad Med Singap.

[17] Sandherr M, Hentrich M, von Lilienfeld-Toal M, Massenkeil G, Neumann S, Penack O (2015). Antiviral prophylaxis in patients with solid tumours and haematological malignancies--update of the guidelines of the infectious diseases working party (AGIHO) of the German Society for Hematology and Medical Oncology (DGHO). Ann Hematol.

[18] Artz AS, Somerfield MR, Feld JJ, Giusti AF, Kramer BS, Sabichi AL (2010). American Society of Clinical Oncology provisional clinical opinion: chronic hepatitis B virus infection screening in patients receiving cytotoxic chemotherapy for treatment of malignant diseases. J Clin Oncol.

[19] Manzano-Alonso ML, Castellano-Tortajada G (2011). Reactivation of hepatitis B virus infection after cytotoxic chemotherapy or immunosuppressive therapy. World J Gastroenterol.

[20] Liaw YF, Kao JH, Piratvisuth T, Chan LY, Chien RN, Liu CJ (2012). Asian-Pacific consensus statement on the management of chronic hepatitis B: a 2012 update. Hepatol Int.

[21] Koh C, Zhao X, Samala N, Sakiani S, Liang TJ, Talwalkar JA (2013). AASLD clinical practice guidelines: a critical review of scientific evidence and evolving recommendations. Hepatology.

[22] Bihl F, Alaei M, Negro F (2010). The new EASL guidelines for the management of chronic hepatitis B infection adapted for Swiss physicians. Swiss Med Wkly.

[23] Chen CY, Huang SY, Cheng A, Chou WC, Yao M, Tang JL (2015). High risk of hepatitis B reactivation among patients with acute myeloid Leukemia. PLoS One.

[24] Nakamura Y, Motokura T, Fujita A, Yamashita T, Ogata E (1996). Severe hepatitis related to chemotherapy in hepatitis B virus carriers with hematologic malignancies. Survey in Japan, 1987-1991. Cancer.

[25] Tassopoulos NC, Papaevangelou GJ, Roumeliotou-Karayannis A, Smedile A, Engle R, Ticehurst JR (1986). Fulminant hepatitis in asymptomatic hepatitis B surface antigen carriers in Greece. J Med Virol.

[26] Kojima H, Abei M, Takei N, Mukai Y, Hasegawa Y, Iijima T (2002). Fatal reactivation of hepatitis B virus following cytotoxic chemotherapy for acute myelogenous leukemia: fibrosing cholestatic hepatitis. Eur J Haematol.

[27] Yujiri T, Tanaka M, Taguchi A, Tanaka Y, Nakamura Y, Tanizawa Y (2014). Reactivation of hepatitis B virus in a hepatitis B surface antigen-negative patient with acute promyelocytic leukemia treated with arsenic trioxide. Ann Hematol.

[28] Mya DH, Han ST, Linn YC, Hwang WY, Goh YT, Tan DC (2012). Risk of hepatitis B reactivation and the role of novel agents and stem-cell transplantation in multiple myeloma patients with hepatitis B virus (HBV) infection. Ann Oncol.

[29] Lee JY, Lim SH, Lee MY, Kim H, Sinn DH, Gwak GY (2015). Hepatitis B reactivation in multiple myeloma patients with resolved hepatitis B undergoing chemotherapy. Liver Int.

[30] Orlando R, Tosone G, Tiseo D, Piazza M, Portella G, Ciancia R (2006). Severe reactivation of hepatitis B virus infection in a patient with hairy cell leukemia: should lamivudine prophylaxis be recommended to HBsAg-negative, anti-HBc-positive patients?. Infection.

[31] Papamichalis P, Alexiou A, Boulbou M, Dalekos GN, Rigopoulou EI (2012). Reactivation of resolved hepatitis B virus infection after immunosuppression: is it time to adopt pre-emptive therapy?. Clin Res Hepatol Gastroenterol.

[32] Huang YH, Hsiao LT, Hong YC, Chiou TJ, Yu YB, Gau JP (2013). Randomized controlled trial of entecavir prophylaxis for rituximab-associated hepatitis B virus reactivation in patients with lymphoma and resolved hepatitis B. J Clin Oncol.

[33] Hsiao LT, Chiou TJ, Gau JP, Yang CF, Yu YB, Liu CY (2015). Risk of reverse Seroconversion of hepatitis B virus surface antigen in Rituximab-treated non-Hodgkin lymphoma patients: a large cohort retrospective study. Medicine (Baltimore).

[34] Demir M, Serin E, Göktürk S, Ozturk NA, Kulaksizoglu S, Ylmaz U (2008). The prevalence of occult hepatitis B virus infection in type 2 diabetes mellitus patients. Eur J Gastroenterol Hepatol.

[35] Wang YY, Lin SY, Sheu WH, Liu PH, Tung KC (2010). Obesity and diabetic hyperglycemia were associated with serum alanine aminotransferase activity in patients with hepatitis B infection. Metabolism.

[36] Kamitsukasa H, Iri M, Tanaka A, Nagashima S, Takahashi M, Nishizawa T (2015). Spontaneous reactivation of hepatitis B virus (HBV) infection in patients with resolved or occult HBV infection. J Med Virol.

